# Risk of fracture among patients with polymyalgia rheumatica and giant cell arteritis: a population-based study

**DOI:** 10.1186/s12916-017-0987-1

**Published:** 2018-01-10

**Authors:** Zoe Paskins, Rebecca Whittle, Alyshah Abdul Sultan, Sara Muller, Milica Blagojevic-Bucknall, Toby Helliwell, Samantha Hider, Edward Roddy, Christian Mallen

**Affiliations:** 1 Arthritis Research UK Primary Care Centre, Research Institute for Primary Care & Health Sciences, Keele University, Staffordshire, ST5 5BG UK; 2 Haywood Academic Rheumatology Centre, Staffordshire and Stoke-on-Trent Partnership Trust, Stoke-on-Trent, Staffordshire, UK

**Keywords:** Fracture, Osteoporosis, Glucocorticoids, Polymyalgia, Giant cell arteritis

## Abstract

**Background:**

Glucocorticoids are associated with increased fracture risk and are the mainstay of treatment in polymyalgia rheumatica (PMR) and giant cell arteritis (GCA). However, fracture risk in these conditions has not been previously quantified. The aim of this study was to quantify the risk of fracture among patients with PMR and GCA.

**Methods:**

A retrospective cohort study was conducted using primary care records from the UK-based Clinical Practice Research Datalink. Individuals aged 40 years and over, with incident diagnoses of PMR or GCA were separately identified from 1990–2004 and followed up until 2015. For each exposed individual, four age-, sex- and practice-matched controls were randomly selected. Incidence rates of fracture per 10,000 person-years were calculated for each disease group and hazard rates were compared to the unexposed using Cox regression models.

**Results:**

Overall, 12,136 and 2673 cases of PMR and GCA, respectively, were identified. The incidence rate of fracture was 148.05 (95% CI 141.16–155.28) in PMR and 147.15 (132.91–162.91) in GCA per 10,000 person-years. Risk of fracture was increased by 63% in PMR (adjusted hazard ratio 1.63, 95% CI 1.54–1.73) and 67% in GCA (1.67, 1.49–1.88) compared to the control populations. Fewer than 13% of glucocorticoid-treated cases were prescribed bisphosphonates.

**Conclusions:**

This study reports, for the first time, a similar increase in fracture risk for patients with PMR and GCA. More needs to be done to improve adherence to guidelines to co-prescribe bisphosphonates. Further research needs to identify whether lower glucocorticoid starting doses and/or aggressive dose reduction reduces fracture risk.

**Electronic supplementary material:**

The online version of this article (doi:10.1186/s12916-017-0987-1) contains supplementary material, which is available to authorized users.

## Background

Polymyalgia rheumatica (PMR) is the most common inflammatory rheumatic disease in older people, whereas giant cell arteritis (GCA) is the most common vasculitis. Collectively, they are the most common indications for long-term (greater than 6 months) glucocorticoid therapy in primary care [1]. The two diseases have a marked overlap between them, with 16–21% of patients with PMR developing GCA during the course of their illness and 40–60% of patients with GCA reporting PMR symptoms [2]. Glucocorticoids remain the first-line recommended treatment for GCA and PMR, and are a well-established cause of osteoporosis and fragility fracture [3]. However, in other inflammatory conditions, increased fracture risk was also observed in patients not using glucocorticoids [4–6], which may support a direct association between inflammation and osteoporosis. Guidelines for the treatment of GCA and PMR recommend early use of methotrexate as a glucocorticoid-sparing agent in patients at increased risk of glucocorticoid adverse effects [7]. Evidence is conflicting as to whether methotrexate has a detrimental effect on bone [8, 9]. However, the finding that cardiovascular events are reduced in patients taking methotrexate [10] suggests that better control of inflammatory burden by the drug may reduce the risk of complications.

Clinical guidelines for PMR management suggest bone protection (bisphosphonate with calcium and vitamin D supplementation) should be considered for all persons aged over 65 years, patients with prior fragility fracture or those requiring higher initial glucocorticoid dose [11], with GCA guidelines suggesting bone protection for all [7].

To date, only one study has reported population-based estimates of fracture risk in GCA in the context of other glucocorticoid associated adverse events, reporting a 1.4-fold increased risk of fracture [12]. This study did not examine the effect of age, glucocorticoid dose or methotrexate on fracture risk and did not report risk at individual fracture sites. There are no population-based estimates of fracture risk in PMR. Other observational studies examining the association between fracture and PMR and/or GCA have been conducted with patients recruited in secondary care [13–16]. Thus, the aim of this study was to separately quantify the risk of fracture among patients with PMR and GCA in a large primary care dataset and assess the impact of age, sex, glucocorticoid and methotrexate on risk.

## Methods

### Data source

We conducted a retrospective cohort study using data from the Clinical Practice Research Datalink (CPRD), a large database containing primary care medical records of 6.9% of the United Kingdom (UK) population, representative of the wider population in terms of age and sex distribution [17]. Practices included in CPRD receive training on recording clinical information, with data from a practice being used only when it has reached a certain standard of quality (up-to-standard; UTS). Patient information is recorded using a coded thesaurus of clinical terms (Read codes).

### Study population

Separate exposed populations were defined for PMR and GCA, which were not mutually exclusive. Patients with PMR and/or GCA, aged 40 and over, were identified based on the presence of one or more relevant Read codes (Additional file 1: Table S1) documented in the patient’s electronic medical record between 1990 and 2004. Each patient was assigned an index date corresponding to the date of their first recorded disease diagnosis. Those diagnosed with PMR or GCA before the study period or within 3 months of their registration with a practice were considered prevalent cases and excluded [18].

Two control populations were separately defined for PMR and GCA. Controls did not have a diagnosis of any inflammatory conditions (PMR, GCA, gout, ankylosing spondylitis, inflammatory bowel disease, rheumatoid arthritis, systemic lupus erythematous or psoriasis) recorded in their entire electronic medical record up until the end of study. For each exposed patient, four controls were randomly selected matched on age, sex and general practice. The non-exposed patient’s index date was defined as their matched exposed patient’s index date.

### Follow-up

The study start date was defined as the date of a patient’s registration with their practice, the date their practice was defined as UTS, the patient’s index date or on January 1, 1990, whichever came latest. The study end date was defined as the earliest of the date of the patient’s death, the date the patient transferred out of the practice, the date of last data collection from that practice, the date of first fracture, or August 31, 2015. Those with less than 12 months UTS data prior to index date and less than 3 years UTS follow-up after the index date were excluded.

### Outcome definition

The event of interest was time from index date until first fracture. As we were concerned with fragility fractures, fractures at four sites encompassing the definition for ‘major osteoporotic fracture’ [19] were selected (vertebrae, humerus, wrist and hip); we also included general codes for fragility fracture where site was unspecified. First fracture was identified using Read codes (Additional file 1: Table S1), which have been previously validated in CPRD [20]. In order to ensure this was the first fracture, patients with a Read code for fracture prior to their index date were excluded.

### Covariates

We extracted information on patient demographics (age, sex) at their index date, lifestyle-related characteristics (body mass index (BMI), smoking status and alcohol consumption) using the measurement nearest to their index date (ever prior to index and up to 1 year after), comorbidities (summarised using the Charlson comorbidity index [21]) and prescription of medications (glucocorticoid, methotrexate, bisphosphonates and proton pump inhibitors (PPIs)) prior to the outcome for both the exposed and non-exposed. BMI was categorised according to the World Health Organization classification as underweight (<18.5 kg/m^2^), normal (18.5–25 kg/m^2^), overweight (25–29.9 kg/m^2^) or obese (≥ 30 kg/m^2^). Those with missing information on BMI, smoking and alcohol use were included as a separate category. Information on falls was collected during the study period. Dose and duration of each glucocorticoid prescription was derived from available information using the algorithm described in Additional file 2, which was subsequently used to calculate the average daily dose. Doses of oral glucocorticoid were converted into a prednisolone-equivalent dosage. Where a condition/prescription is present in the record, we assume that the patient received this diagnosis/prescription at the time it was recorded. Where there is no record of a condition/prescription, it is assumed that the patient did not have this condition/prescription.

### Statistical analysis

All data summaries and analyses were performed separately for the PMR and GCA cohorts. Demographics and lifestyle-related characteristics were summarised using frequencies and percentages. Incidence rates were expressed as the number of first fractures per 10,000 person years. To assess the association between exposure and time to osteoporotic fracture, Cox proportional hazard models were used to obtain estimates of hazard ratios (HRs) with 95% confidence intervals (CIs), based on robust standard errors to account for matching. Unadjusted estimates were obtained followed by adjustment for age, sex, BMI, alcohol, smoking, Charlson comorbidity index and PPI use. To avoid over-adjustment, models were only adjusted for confounding factors (associated with both the outcome and exposure) which affected estimates by > 10%. Proportionality of hazards assumption was tested throughout using Schoenfeld residuals. Subgroup analyses by sex and age group were performed. The timing of fracture reporting in relation to PMR and GCA diagnosis was then assessed by comparing the absolute rate of fracture at yearly intervals up to 5 years after PMR/GCA diagnosis among the exposed and non-exposed in terms of incidence rate ratios using a Poisson regression model. Analyses were stratified by fracture site. The effect of the use of methotrexate and the cumulative dose of glucocorticoid on the incidence of fracture was evaluated by estimating hazard ratios within those with PMR/GCA, excluding the controls without PMR/GCA from this analysis. Any methotrexate use was compared to none and each quintile of average daily glucocorticoid dose was compared to lowest quintile. The analyses were adjusted as above, with the addition of bisphosphonate use.

Sensitivity analyses were performed, defining the exposure status as a Read code for PMR or GCA, plus two or more prescriptions for glucocorticoids during the study period; hence, patients who did not have two or more glucocorticoid prescriptions were excluded, along with their matched controls.

Patients with GCA may have symptoms of both PMR and GCA, whereas patients with PMR would not be expected to have symptoms of GCA; therefore, additional sensitivity analysis was performed estimating the risk of fracture in the group of patients with PMR diagnosis, excluding those with GCA codes. Finally, analyses considering complete cases only (no missing category for smoking, alcohol use and BMI) were performed as sensitivity analyses and compared to the main results.

All analyses were performed using Stata/MP 14.2 (Stata Corporation, TX, USA).

## Results

### Basic characteristics

The study included 12,136 PMR and 2673 GCA patients, individually matched to 46,238 and 10,423 non-exposed patients (controls), respectively (Table 1). Of these, 735 patients were coded as having both conditions (6.1% and 27.5% of the PMR and GCA group, respectively). The median follow-up for PMR and GCA patients was 9 years, corresponding to 114,082 and 25,212 person-years of follow-up, respectively. Compared to controls, PMR and GCA patients had higher BMI (> 30 kg/m^2^; PMR: 32.6% vs. 29.0%; GCA: 15.6% vs. 13.6%). GCA, but not PMR, patients were more likely to smoke than their matched controls (GCA: 18.7% vs. 14.0%; PMR: 12.9% vs. 13.1%) and patients with both conditions were more likely to have consulted primary care for a fall within the study period compared to their matched controls (PMR: 31.1% vs. 24.9%; GCA: 31.8% vs. 25.1%). As expected, PPI, bisphosphonate and methotrexate prescriptions were more common among PMR and GCA patients compared to controls. Approximately 88% of PMR patients and 83% of GCA patients had at least two glucocorticoid prescriptions post-diagnosis.

**Table 1:** Basic characteristics of polymyalgia rheumatica (PMR) exposed/non-exposed and giant cell arteritis (GCA) exposed/non-exposed, stratified by steroid use in the 1-year period prior to outcome

	PMR		GCA	
Variables	Present *N* = 12,136 *n* (%)	Non-present *N* = 46,238 *n* (%)	Present *N* = 2673 *n* (%)	Non-present *N* = 10,423 *n* (%)
Follow up time; median (IQR)	9.27 (5.74–12.36)	9.59 (5.97–12.61)	9.13 (5.87–12.38)	9.97 (6.23–13.00)
Total fractures	1689 (13.92)	5173 (11.19)	371 (13.88)	1,142 (10.96)
Fracture site				
Wrist	464 (27.47)	1568 (30.31)	118 (31.81)	356 (31.17)
Vertebra	276 (16.34)	482 (9.32)	72 (19.41)	123 (10.77)
Humerus	220 (13.03)	693 (13.40)	35 (9.43)	145 (12.70)
Hip	563 (33.33)	1844 (35.65)	115 (31.00)	419 (36.69)
Other	166 (9.83)	586 (11.33)	31 (8.36)	99 (8.67)
Male, n (%)	3729 (30.73)	14,078 (30.45)	774 (28.96)	2981 (28.60)
Mean age (SD)	72.04 (9.70)	71.49 (9.52)	71.12 (9.91)	70.69 (9.76)
Body mass index, kg/m^2^				
< 18.5	161 (1.33)	676 (1.46)	42 (1.57)	140 (1.34)
18.5–25	3723 (32.55)	14,031 (30.35)	795 (29.74)	3102 (29.76)
25–30	3950 (32.55)	13,401 (28.98)	844 (31.58)	2941 (28.22)
> 30	1919 (15.81)	6200 (13.41)	417 (15.60)	1412 (13.55)
Missing	2383 (19.64)	11,930 (25.80)	575 (21.51)	2828 (27.13)
Current smoker				
Yes	1568 (12.92)	6045 (13.07)	500 (18.71)	1454 (13.95)
No	9286 (76.52)	32,579 (70.46)	1858 (69.51)	7111 (68.22)
Missing	1282 (10.56)	7614 (16.47)	315 (11.78)	1858 (17.83)
Alcohol (units per week)				
Never/Ex-drinker	2972 (24.49)	10,000 (21.63)	708 (26.49)	2329 (22.34)
1–9	3285 (27.07)	12,087 (26.14)	705 (26.37)	2589 (24.84)
≥ 10	1453 (11.97)	5247 (11.35)	275 (10.29)	1105 (10.60)
Missing	4426 (36.47)	18,904 (40.88)	985 (36.85)	4400 (42.21)
Consultation for falls	3771 (31.07)	11,504 (24.88)	849 (31.76)	2612 (25.06)
Charlson comorbidity index; median (IQR)	3 (1–4)	1 (0–3)	2 (1–4)	1 (0–3)
Proton pump inhibitors	7715 (63.57)	20,710 (44.79)	1749 (65.43)	4670 (44.80)
Bisphosphonate	1479 (12.19)	829 (1.79)	413 (15.45)	194 (1.86)
Methotrexate	706 (5.82)	211 (0.46)	122 (4.56)	53 (0.51)
> 1 Glucocorticoid prescription after index	10,738 (88.48)	5894 (12.75)	2232 (83.50)	1443 (13.84)
Glucocorticoids treatment length; Median months (IQR)	15.74 (6.51–34.36)	0.99 (0.23–3.07)	12.93 (4.01–32.49)	1.05 (0.23–5.13)

### Risk of fracture

A total of 1689 (13.9%) patients in the PMR group and 371 (14%) in the GCA group experienced a fracture, corresponding to incidence rates of 148 (95% CI 141–155) and 147 (133–163) per 10,000 person-years, respectively (Table 2). After adjustment, the risk of fracture was 63% higher in patients with PMR (adjusted HR (aHR) 1.63, 95% CI 1.54–1.73) and 67% higher in those with GCA (aHR 1.67, 95% CI 1.49–1.88) compared to their separate control populations. Whilst the incident rate of fracture was higher in women than men, in both the PMR and GCA cases, the relative increased risk compared to controls was similar for both sexes. Among those aged 50–60 years, the risk of fracture was more than twice as high in patients with PMR or GCA compared to their controls (PMR: aHR 2.23, 95% CI 1.79–2.79; GCA: aHR 2.27, 95% CI 1.25–4.15). This effect was less pronounced at older ages (test for trends *P* < 0.001).

**Table 2:** Incidence rates and hazard ratios (HRs) for associations of fracture with exposure to polymyalgia rheumatica (PMR) and giant cell arteritis (GCA)

Variables	Present		Non-present		Unadjusted HR (95% CI)	Adjusted HR (95% CI)^a^
	Number with fracture	Rate per 10,000 person-years (95% CI)	Number with fracture	Rate per 10,000 person-years		
PMR						
Overall	1689	148.05 (141.16–155.28)	5173	115.67 (112.56–118.87)	1.29 (1.22–1.36)	1.63 (1.54–1.73)
*Sex*						
Male	244	68.79 (60.68–77.99)	707	52.07 (48.37–56.06)	1.32 (1.15–1.53)	1.54 (1.33–1.81)
Female	1445	183.81 (174.57–193.54)	4466	143.4 (139.25–147.67)	1.29 (1.22–1.37)	1.61 (1.52–1.72)
*Age in years*						
40–50	16	45.58 (27.93–74.41)	48	32.55 (24.53–43.2)	1.40 (0.81–2.41)	1.92 (1.01–3.69)
50–60	116	81.29 (67.77–97.52)	264	46.04 (40.81–51.94)	1.78 (1.43–2.2)	2.23 (1.79–2.79)
60–70	357	112.33 (101.26–124.61)	1018	78.56 (73.88–83.53)	1.44 (1.28–1.62)	1.85 (1.56–2.10)
70–80	753	161.12 (150.01–173.04)	2555	138.51 (133.25–143.99)	1.17 (1.08–1.26)	1.51 (1.38–1.64)
≥ 80	447	251.34 (229.08–275.75)	1288	210.89 (199.68–222.72)	1.21 (1.08–1.34)	1.61 (1.43–1.81)
GCA						
Overall	371	147.15 (132.91–162.91)	1142	109.52 (103.35–116.06)	1.36 (1.22–1.53)	1.67 (1.49–1.88)
*Sex*						
Male	49	66.62 (50.35–88.15)	127	42.37 (35.61–50.42)	1.59 (1.15–2.2)	1.62 (1.17–2.24)
Female	322	180.31 (161.66–201.12)	1015	136.6 (128.45–145.27)	1.34 (1.19–1.51)	1.67 (1.47–1.90)
*Age in years*						
40–50	4	35.23 (13.22–93.86)	9	18.87 (9.82–36.26)	1.87 (0.63–5.58)	1.98 (0.66–5.94)
50–60	18	55.2 (34.78–87.61)	46	34.94 (26.17–46.65)	1.56 (0.9–2.71)	2.27 (1.25–4.15)
60–70	85	112.74 (91.15–139.45)	257	78.19 (69.19–88.36)	1.49 (1.17–1.89)	1.79 (1.40–2.30)
70–80	181	185.8 (160.61–214.94)	579	141.96 (130.85–154)	1.34 (1.13–1.57)	1.65 (1.39–1.95)
≥ 80	83	234.75 (189.31–291.1)	251	197.88 (174.85–223.94)	1.20 (0.93–1.54)	1.48 (1.13–1.94)

^a^Adjusted for confounding factors which affected the HR > 10%, from age, sex, BMI, alcohol consumption, smoking status, Charlson comorbidity index and PPI use when not stratified by those

**Fig. 1:**
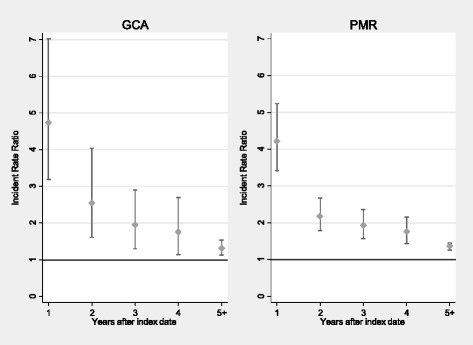
Incident rate ratios of fracture in GCA and PMR populations. Adjusted for confounding factors which affect the HR > 10%, from age, sex, BMI, alcohol consumption, smoking status, Charlson comorbidity index and PPI use

Figure 1 shows incidence rate ratio of fracture in the years following PMR and GCA diagnosis compared to controls. For patients with both conditions, the incidence rate ratio of fracture remained significantly increased 5 years post-diagnosis.

### Risk by fracture site

There was an increased risk of fractures at all sites in PMR patients compared to controls. However, in GCA, there was an increased risk of wrist, vertebra and hip fractures, but not of humeral fracture (Table 3). In both conditions, the risk was greatest for vertebral fractures (PMR: aHR 2.57, 95% CI 2.19–3.02; GCA: aHR 2.97, 95% CI 2.21–3.99).

**Table 3:** Incidence rates and hazard ratios (HRs) for associations of site-specific fracture with exposure to polymyalgia rheumatica (PMR) and giant cell arteritis (GCA)

Variables	Present		Non-present		Unadjusted HR (95% CI)	Adjusted HR (95% CI)^a^
	Number with fracture	Rate per 10,000 person-years (95% CI)	Number with fracture	Rate per 10,000 person-years		
PMR						
Wrist	464	40.67 (37.14–44.55)	1568	35.06 (33.37–36.84)	1.16 (1.05–1.29)	1.70 (1.52–1.90)
Vertebra	276	24.19 (21.50–27.22)	482	10.78 (9.86–11.78)	2.25 (1.94–2.61)	2.57 (2.19–3.02)
Humerus	220	19.28 (16.90–22.01)	693	15.50 (14.38–16.69)	1.25 (1.07–1.45)	1.63 (1.39–1.91)
Hip	563	49.35 (45.44–53.60)	1844	41.23 (39.39–43.16)	1.20 (1.10–1.32)	1.45 (1.31–1.60)
Unspecified	166	14.55 (12.50–16.94)	586	13.10 (12.08–14.21)	1.14 (0.96–1.35)	1.26 (1.06–1.51)
GCA						
Wrist	118	46.80 (39.08–56.06)	356	34.14 (30.77–37.88)	1.37 (1.12–1.68)	1.84 (1.49–2.28)
Vertebra	72	28.56 (22.67–35.98)	123	11.80 (9.88–14.08)	2.44 (1.84–3.23)	2.97 (2.21–3.99)
Humerus	35	13.88 (9.97–19.33)	145	13.91 (11.82–16.36)	1.01 (0.70–1.45)	1.19 (0.81–1.73)
Hip	115	45.61 (37.99–54.76)	419	40.18 (36.51–44.22)	1.16 (0.95–1.42)	1.39 (1.13–1.72)
Unspecified	31	12.30 (8.65–17.48)	99	9.49 (7.80–11.56)	1.37 (0.91–2.05)	1.32 (0.87–2.00)

^a^Adjusted for confounding factors which affected the HR > 10%, from age, sex, BMI, alcohol consumption, smoking status, Charlson comorbidity index and PPI use

### Risk by prescriptions

Overall, 122 (4.6%) of patients with GCA and 706 (5.8%) of those with PMR received a methotrexate prescription. There were no significant differences in risk of fracture between those prescribed, or not, methotrexate in either group (PMR: aHR 0.96, 95% CI 0.75–1.21, GCA: aHR 1.36, 95% CI 0.84–2.19) (Table 4). Patients with PMR and GCA received glucocorticoids for a median of 16 months (IQR 7–34) and 13 months (IQR 4–33), respectively. In both conditions, patients in the highest quintile of glucocorticoid average daily dose had a higher risk of fracture compared to the lowest quintile (PMR: aHR 1.85, 95% CI 1.59–2.17; GCA: aHR 2.09, 95% CI 1.46–2.99). Among GCA and PMR patients with at least two prescriptions for glucocorticoids, only 12.6% and 10.1%, respectively, were prescribed bisphosphonates.

**Table 4:** Incident rates and hazard ratios (HRs) for association of fracture with methotrexate and glucocorticoid use among those with polymyalgia rheumatica (PMR) and giant cell arteritis (GCA)

Variables	Number of patients	Number with fracture	Rate per 10,000 person-years (95% CI)	Unadjusted HR (95% CI)	Adjusted HR (95% CI)^a^
PMR					
*Methotrexate*					
No	11,430	1614	151.32 (144.11–158.89)	Reference	Reference
Yes	706	75	101.03 (80.57–126.69)	0.65 (0.52–0.83)	0.96 (0.75–1.21)
*Glucocorticoid average daily dose (quintiles)*					
Quintile 1 (1–4.9 mg)	2148	281	135.72 (120.74–152.55)	Reference	Reference
Quintile 2 (4.9–6.1 mg)	2148	286	139.86 (124.56–157.05)	1.03 (0.88–1.22)	1.12 (0.95–1.32)
Quintile 3 (6.1–7.5 mg)	2147	291	141.52 (126.16–158.75)	1.04 (0.88–1.23)	1.16 (0.99–1.37)
Quintile 4 (7.5–10.0 mg)	2148	283	141.32 (125.78–158.78)	1.04 (0.88–1.23)	1.27 (1.08–1.50)
Quintile 5 (10.0–73.3 mg)	2147	367	196.70 (177.57–217.89)	1.46 (1.25–1.71)	1.85 (1.59–2.17)
GCA					
*Methotrexate*					
No	2551	353	147.51 (132.90–163.73)	Reference	Reference
Yes	122	18	140.38 (88.45–222.82)	0.94 (0.58–1.50)	1.36 (0.84–2.19)
*Glucocorticoid average daily dose (quintiles)*					
Quintile 1 (1.1–5.7 mg)	447	52	118.06 (80.96–154.93)	Reference	Reference
Quintile 2 (5.7–7.5 mg)	446	63	147.64 (115.33–188.99)	1.26 (0.87–1.81)	1.51 (1.05–2.19)
Quintile 3 (7.5–9.8 mg)	447	59	138.05 (106.85–178.18)	1.17 (0.81–1.70)	1.46 (1.00–2.12)
Quintile 4 (9.8–13.5 mg)	446	57	138.33 (106.70–179.34)	1.18 (0.81–1.71)	1.58 (1.08–2.30)
Quintile 5 (13.5–83.9 mg)	446	72	181.18 (143.81–228.25)	1.54 (1.08–2.20)	2.09 (1.46–2.99)

^a^Adjusted for confounding factors which affected the HR > 10%, from age, sex, BMI, alcohol consumption, smoking status, bisphosphonate use, Charlson comorbidity index and PPI use. Patients not on glucocorticoids were excluded from this analysis

### Sensitivity analyses

Restricting analyses to the cases with two or more prescriptions for glucocorticoids did not alter the overall adjusted estimates of fracture significantly (GCA: HR 1.59, 95% CI 1.40–1.81; PMR: HR 1.62, 95% CI 1.53–1.73).

The risk of fracture was similar in PMR-only cases, i.e. when cases with any GCA diagnoses were removed (PMR only: HR 1.61, 95% CI 1.52–1.71).

When analysing complete cases only, results were similar to when including a missing category for smoking, alcohol use and BMI (results not presented).

## Discussion

This is the first study to quantify the risk of fracture in people with PMR and/or GCA, demonstrating an increased risk of fracture of 63% in PMR and 67% in GCA when compared with age-, sex- and practice-matched controls. The increased risk was similar in men and women, and highest for vertebral fractures, followed by wrist fractures. We found that risk of fracture is highest within the first year of diagnosis and remains significantly elevated for more than 5 years after diagnosis. In both conditions, fracture risk was higher in those who received a higher average daily dose of glucocorticoids than in those who received a lower daily dose. The median duration of steroid use was less than 15 months for both conditions, although 25% of patients with PMR had more than 34 months of treatment. Less than 13% of glucocorticoid-treated patients had ever received bone protection with a bisphosphonate. The use of methotrexate was not associated with an increased risk of fracture.

### Strengths and limitations of the study

By utilising CPRD and selecting all incident cases of PMR and GCA during the study period, our findings are generalisable to the wider UK primary care population. Matching cases and controls by age, sex and practice reduces the likelihood of confounding. However, as in any database study, it is possible that residual confounding remains. Furthermore, our methods do not facilitate the identification of asymptomatic or undiagnosed vertebral fractures. Vertebral fractures often do not come to clinical attention [22], and it is theoretically possible that the pain of GCA and/or PMR may mask the presentation of these fractures; both of these factors may result in underestimation of this risk. A number of further limitations of our study are worthy of acknowledgement. First, we relied on General Practitioner diagnoses of GCA and PMR. The diagnosis of GCA has previously been validated in CPRD [23], although PMR has not. Other conditions may be misclassified as PMR, which may explain why 12% of those with a PMR Read code were not treated with two or more glucocorticoid prescriptions. However, a sensitivity analysis which excluded these participants did not change our findings, providing confidence in our estimates. Second, we excluded patients without 3 years of UTS follow-up records after baseline, which may have resulted in the cases being healthier and a potentially bias in our findings towards the null hypothesis. Third, we cannot account for drugs prescribed in secondary care, for example, parenteral osteoporosis treatments and intravenous or intramuscular glucocorticoids. However, in practice, oral glucocorticoids remain the mainstay of treatment, intravenous glucocorticoids are more likely to be prescribed to those already on high oral doses, intramuscular doses are unlikely to be prescribed exclusively in secondary care, and the proportion of patients on parenteral osteoporosis treatment is small. Further, patients with GCA and PMR may have consulted more frequently, resulting in ascertainment bias, particularly relevant to the identification of vertebral fractures which often go undiagnosed [22]. However, adjustment for consultation rate made little difference to the results. Finally, it was not possible to determine the inflammatory burden associated with our conditions of interest. It is theoretically possible that the inflammatory component of GCA is more deleterious to bones than glucocorticoids, and that when glucocorticoids effectively treat high levels of inflammation, the fracture risk incurred by the glucocorticoids themselves is offset.

### Comparison with other studies

Previous studies have demonstrated a dose-dependent increase in fracture risk with glucocorticoid dose [3] and these findings were mirrored in our study. However, previous studies of fracture risk in patients treated with glucocorticoids in CPRD suggest a rapid offset of increased risk on treatment cessation, with most excess risk disappearing within 1 year of stopping [3]. In our study, risk persisted for more than 5 years after diagnosis, but we did not compare rates before and after stopping. A previous study using CPRD found a much lower relative risk (1.09) of wrist fracture in glucocorticoid users [3]; higher rates of wrist fractures in our study may have been due to the increased number of falls recorded during the study period. Rossini et al. [13] found that vertebral fractures were more common than fractures at other sites in patients with GCA; however, theirs was an observational study in which patients had radiographs that would have resulted in an increased detection of subclinical fractures in the spine. The previous population study examining fracture risk in GCA found a higher risk of fracture in men than women, which is in contrast to our findings [12]. This may be related to the inclusion of fractures at sites that are typically traumatic, as opposed to fragility fractures. Other studies have found much higher rates of bisphosphonate use; in our study, the proportion of patients prescribed bisphosphonates was half of that in previous prospective primary care cohorts of patients with PMR [24] or prescribed glucocorticoid generally [25], and one-sixth of the proportion of patients with PMR treated with bisphosphonates in secondary care [26]. This might be explained by changes in practice over the dates of study inclusion, although UK guidance in 2002 advocated the use of bisphosphonates in patients treated with > 7.5 mg prednisolone aged 65 and over [27].

The finding that fracture risk in PMR and GCA is similar is surprising given that considerably lower dose glucocorticoids are recommended for PMR than for GCA. If the UK guidelines for typical dosing and duration of glucocorticoid are followed, a patient with PMR would be expected to receive half the cumulative dose of steroids as compared to the cumulative dose a patient with GCA would be expected to receive (2817.5 mg over 15 months compared with 5705 mg over 18 months) [28, 29]. Relatively little is known about ‘real life’ steroid use in PMR and GCA; however, our findings demonstrate that, PMR is, in practice, treated for a longer duration than GCA. Furthermore, in PMR, which is more likely to be treated exclusively by primary care physicians than secondary care specialists (especially in the UK where our study was based), daily doses are also likely to be higher than those recommended; in a UK survey of 1249 randomly selected General Practitioners, over 40% reported initiating doses of 30 mg of prednisolone or more, in excess of the EULAR/ACR recommended starting dose of between 15 and 25 mg [30, 31].

## Conclusion

Our study has three important implications for clinical practice. First, the bisphosphonate use in this study was low and more needs to be done to improve adherence to current guidelines. Second, it highlights that clinicians need to be mindful of falls risk in this population. The persisting risk of fracture at 5 years and the higher risk of wrist fractures seen herein may be explained by an increase in falls observed during the study period. Third, it raises important questions about the doses of glucocorticoids used to treat PMR. We therefore suggest more research is needed into optimal glucocorticoid tapering regimes [11] to identify whether lower starting doses and/or aggressive dose reduction reduces fracture risk, particularly in PMR, and to explore the safety and benefit of using non-glucocorticoid treatments and glucocorticoid sparing agents in the treatment of these conditions.

## Additional files

Additional file 1: Table S1. Read codes for exposure and outcome definition. (DOCX 13 kb)

Additional file 2: Algorithm for deriving glucocorticoid average dose and duration. (DOCX 22 kb)

## Abbreviations

aHR: adjusted hazard ratio
BMI: body mass index
CPRD: Clinical Practice Research datalink
GCA: giant cell arteritis
HR: hazard ratio
IQR: interquartile range
PMR: polymyalgia rheumatica
PPI: proton pump inhibitor
UK: United Kingdom
UTS: up to standard
