# Supplementary material for: Risk of fracture among patients with polymyalgia rheumatica and giant cell arteritis: a population-based study
Source: BMC Med. 2018 Jan 10;16:4. doi: 10.1186/s12916-017-0987-1 (PMC5761155; doi:10.1186/s12916-017-0987-1)
Supplement: Supplementary file 1 — Read codes for exposure and outcome definition. (DOCX 13 kb) [file 12916_2017_987_MOESM1_ESM.docx]

Supplementary Table 1: Read codes for exposure and outcome definition

Exposure: PMR: N200.00 GCA with PMR; N20..11 polymyalgia; N20..00 PMR; GCA: G755100 temporal arteritis; G755200 Horton’s disease; G755z00 GCA NOS; G755.00 GCA; G755000 cranial arteritis; N200.00 GCA with PMR; Nyu4100 other GCA

Outcome:

| **Read code** | **Read term** |
| --- | --- |
| Syu5400 | [X]Fracture of forearm, unspecified |
| Syu6500 | [X]Fracture of other & unspecified parts of wrist and hand |
| Syu5300 | [X]Fracture of other parts of forearm |
| Syu4300 | [X]Fracture of other parts of shoulder and upper arm |
| Syu4400 | [X]Fracture of shoulder and upper arm, unspecified |
| NyuB000 | [X]Other osteoporosis with pathological fracture |
| NyuB800 | [X]Unspecified osteoporosis with pathological fracture |
| S2...11 | Arm fracture |
| S234A00 | Closd dorsal Barton's fracture |
| S234100 | Closed Colles' fracture |
| S234A12 | Closed dorsal Barton fracture-subluxation |
| S234A11 | Closed dorsal Barton's fracture-dislocation |
| S130.00 | Closed fracture acetabulum |
| S234D00 | Closed fracture distal radius, extra-articular, other type |
| S234C00 | Closed fracture distal radius, intra-articular, die-punch |
| S234E00 | Closed fracture distal radius, intra-articular, other type |
| S104.00 | Closed fracture lumbar vertebra |
| S104000 | Closed fracture lumbar vertebra, burst |
| S104500 | Closed fracture lumbar vertebra, posterior arch |
| S104300 | Closed fracture lumbar vertebra, spinous process |
| S104400 | Closed fracture lumbar vertebra, transverse process |
| S104600 | Closed fracture lumbar vertebra, tricolumnar |
| S104100 | Closed fracture lumbar vertebra, wedge |
| S302011 | Closed fracture of femur, greater trochanter |
| S302400 | Closed fracture of femur, intertrochanteric |
| S302012 | Closed fracture of femur, lesser trochanter |
| S310.00 | Closed fracture of femur, shaft or unspecified part |
| S300y11 | Closed fracture of femur, subcapital |
| S300A00 | Closed fracture of femur, upper epiphysis |
| S234z00 | Closed fracture of forearm, lower end, NOS |
| S23x000 | Closed fracture of forearm, unspecified |
| S222000 | Closed fracture of humerus NOS |
| S222100 | Closed fracture of humerus, shaft |
| S222.00 | Closed fracture of humerus, shaft or unspecified part |
| S222z00 | Closed fracture of humerus, shaft or unspecified part NOS |
| S220500 | Closed fracture of humerus, upper epiphysis |
| S114.00 | Closed fracture of lumbar spine with spinal cord lesion |
| S30y.00 | Closed fracture of neck of femur NOS |
| S302.00 | Closed fracture of proximal femur, pertrochanteric |
| S220z00 | Closed fracture of proximal humerus not otherwise specified |
| S220200 | Closed fracture of proximal humerus, anatomical neck |
| S220000 | Closed fracture of proximal humerus, unspecified part |
| S23x100 | Closed fracture of radius (alone), unspecified |
| S234.00 | Closed fracture of radius and ulna, lower end |
| S23xz00 | Closed fracture of radius and ulna, NOS |
| S232z00 | Closed fracture of radius and ulna, shaft, NOS |
| S23x.00 | Closed fracture of radius and ulna, unspecified part |
| S232000 | Closed fracture of radius, shaft, unspecified |
| S30..00 | Fracture of neck of femur |
| S23..00 | Fracture of radius and ulna |
| S23z.00 | Fracture of radius and ulna, NOS |
| S23x111 | Fracture of radius NOS |
| S30y.11 | Hip fracture NOS |
| N331600 | Idiopathic osteoporosis with pathological fracture |
| N331N11 | Minimal trauma fracture |
| N331M11 | Minimal trauma fracture due to unspecified osteoporosis |
| S31..00 | Other fracture of femur |
| S102y00 | Other specified closed fracture thoracic vertebra |
| N331B00 | Postmenopausal osteoporosis with pathological fracture |
| S234.11 | Wrist fracture - closed |
| SR11.00 | Fractures involving thorax with lower back and pelvis |
| N331N00 | Fragility fracture |
| N331M00 | Fragility fracture due to unspecified osteoporosis |
| S234F00 | Closed Barton's fracture |
| S104200 | Closed fracture lumbar vertebra, spondylolysis |
| S312200 | Closed fracture of femur, lower epiphysis |
| S310000 | Closed fracture of femur, unspecified part |
| S234000 | Closed fracture of forearm, lower end, unspecified |
| S232.00 | Closed fracture of radius and ulna, shaft |
| S23x300 | Closed fracture of the radius and ulna |
| S102100 | Closed fracture thoracic vertebra, wedge |
| S228.00 | Fracture of lower end of humerus |
| S15..00 | Fracture of thoracic vertebra |
| S30..11 | Hip fracture |
| S10B600 | Multiple fractures of lumbar spine and pelvis |
| S150.00 | Multiple fractures of thoracic spine |
| N331800 | Osteoporosis + pathological fracture lumbar vertebrae |
| N331900 | Osteoporosis + pathological fracture thoracic vertebrae |
| N331K00 | Collapse of thoracic vertebra due to osteoporosis |
| N331L00 | Collapse of vertebra due to osteoporosis NOS |
| N331.14 | Osteoporotic vertebral collapse |
| S11x.00 | Closed fracture of spine with spinal cord lesion unspecified |
| S10x.00 | Closed fracture of spine, unspecified, |
| S234200 | Closed fracture of the distal radius, unspecified |
| S220.00 | Closed fracture of the proximal humerus |
| S232100 | Closed fracture of the radial shaft |
| S232200 | Closed fracture of the ulnar shaft |
| S112z00 | Closed fracture of thoracic spine with cord lesion NOS |
| S112.00 | Closed fracture of thoracic spine with spinal cord lesion |
| S30w.00 | Closed fracture of unspecified proximal femur |
| S300300 | Closed fracture proximal femur, basicervical |
| S302100 | Closed fracture proximal femur, intertrochanteric, two part |
| S300200 | Closed fracture proximal femur, midcervical section |
| S300y00 | Closed fracture proximal femur, other transcervical |
| S300600 | Closed fracture proximal femur, subcapital, Garden grade I |
| S300700 | Closed fracture proximal femur, subcapital, Garden grade II |
| S300800 | Closed fracture proximal femur, subcapital, Garden grade III |
| S300900 | Closed fracture proximal femur, subcapital, Garden grade IV |
| S302200 | Closed fracture proximal femur, subtrochanteric |
| S300.00 | Closed fracture proximal femur, transcervical |
| S300z00 | Closed fracture proximal femur, transcervical, NOS |
| S300100 | Closed fracture proximal femur, transepiphyseal |
| S220700 | Closed fracture proximal humerus, four part |
| S220300 | Closed fracture proximal humerus, greater tuberosity |
| S220400 | Closed fracture proximal humerus, head |
| S220100 | Closed fracture proximal humerus, neck |
| S220600 | Closed fracture proximal humerus, three part |
| S234B00 | Closed fracture radial styloid |
| S234600 | Closed fracture radius and ulna, distal |
| S232300 | Closed fracture radius and ulna, middle |
| S230600 | Closed fracture radius, head |
| S230700 | Closed fracture radius, neck |
| S102.00 | Closed fracture thoracic vertebra |
| S102z00 | Closed fracture thoracic vertebra not otherwise specified |
| S102000 | Closed fracture thoracic vertebra, burst |
| S102500 | Closed fracture thoracic vertebra, posterior arch |
| S102300 | Closed fracture thoracic vertebra, spinous process |
| S102200 | Closed fracture thoracic vertebra, spondylolysis |
| S102400 | Closed fracture thoracic vertebra, transverse process |
| S300311 | Closed fracture, base of neck of femur |
| S4C2.00 | Closed fracture-subluxation of the wrist |
| S4C2100 | Closed fracture-subluxation radiocarpal joint |
| S4C2000 | Closed fracture-subluxation, distal radio-ulnar jt |
| S234800 | Closed Galeazzi fracture |
| S150000 | Closed multiple fractures of thoracic spine |
| S114100 | Closed spinal fracture with complete lumbar cord lesion |
| S114000 | Closed spinal fracture with unspecified lumbar cord lesion |
| S234912 | Closed volar Barton fracture-subluxation |
| S234900 | Closed volar Barton's fracture |
| S234911 | Closed volar Barton's fracture-dislocation |
| S112700 | Cls spinal fracture with complete thorac cord lesion, T7-12 |
| S112A00 | Cls spinal fracture with posterior thorac cord lesion, T7-12 |
| S112600 | Cls spinal fracture with unspec thoracic cord lesion, T7-12 |
| S112000 | Cls spinal fracture with unspec thoracic cord lesion,T1-6 |
| S112100 | Cls spinal fracture wth complete thoracic cord lesion,T1-6 |
| N331500 | Drug-induced osteoporosis with pathological fracture |
| N1y1.00 | Fatigue fracture of vertebra |
| S242.00 | Fracture at wrist and hand level |
| S31z.00 | Fracture of femur, NOS |
| S22..00 | Fracture of humerus |
| S22z.00 | Fracture of humerus NOS |
| S23C.00 | Fracture of lower end of both ulna and radius |
| S23B.00 | Fracture of lower end of radius |
| S10B.00 | Fracture of lumbar spine and pelvis |
| S10B000 | Fracture of lumbar vertebra |
| S314.00 | Fracture of shaft of femur |
| S227.00 | Fracture of shaft of humerus |
| S239.00 | Fracture of shaft of radius |
| S23A.00 | Fracture of shafts of both ulna and radius |
| S11..00 | Fracture of spine with spinal cord lesion |
| S11z.00 | Fracture of spine with spinal cord lesion NOS |
| S10..00 | Fracture of spine without mention of spinal cord injury |
| S10z.00 | Fracture of spine without mention of spinal cord lesion NOS |
| S226.00 | Fracture of upper end of humerus |
| S11..12 | Fracture of vertebra with spinal cord lesion |
| S10..12 | Fracture of vertebra without spinal cord lesion |
| 14G8.00 | H/O: vertebral fracture |
| N331.14 | Osteoporotic vertebral collapse |
| N331.12 | Collapse of vertebra NOS |
| N331F00 | Collapse of thoracic vertebra |
| N331G00 | Collapse of lumbar vertebra |
| N331K00 | Collapse of thoracic vertebra due to osteoporosis |
| N331J00 | Collapse of lumbar vertebra due to osteoporosis |
| N331L00 | Collapse of vertebra due to osteoporosis NOS |
| N331D00 | Collapsed vertebra NOS |
| N331H00 | Collapse of cervical vertebra due to osteoporosis |
| N331.14 | Osteoporotic vertebral collapse |
| N331111 | Collapse of lumbar vertebra |
| N331011 | Collapse of thoracic vertebra |
| Nyu6700 | [X]Collapsed vertebra in diseases classified elsewhere |
| N331.11 | Collapse of spine NOS |
