# Supplementary material for: Risk of fracture among patients with polymyalgia rheumatica and giant cell arteritis: a population-based study
Source: BMC Med. 2018 Jan 10;16:4. doi: 10.1186/s12916-017-0987-1 (PMC5761155; doi:10.1186/s12916-017-0987-1)
Supplement: Supplementary file 2 — Algorithm for deriving glucocorticoid average dose and duration. (DOCX 22 kb) [file 12916_2017_987_MOESM2_ESM.docx]

Algorithm for deriving GC average dose and duration

- Missing prescription quantities (number of tablets prescribed) were first imputed with the average quantity of other prescriptions for the same drug and strength
- Prescription duration was replaced with the time difference between prescription dates if two prescriptions were within 90 days
- If there were two prescriptions on the same day, both prescription durations were replaced as the time till next prescription (on a different day)
- If the quantity was less than the prescription duration, the prescription duration was changed to equal the quantity
- If prescription duration was still missing, or within 7 days of another prescription for a different drug/strength, the duration was replaced with quantity divided by numerical daily dose if the quantity is greater than numerical daily dose and numerical daily dose isn’t missing
- Is duration was still missing, it was replaced with the average of that patient’s duration for their other prescriptions of the same drug with the same strength
- If duration was still missing, it was replaced with the average duration for all other patient’s prescriptions of the same drug with the same strength
- If the prescription duration was more than 90 days, it was replaced as 90 days
- If the quantity was less than the numerical daily dose, the quantity was replaced to equal the numerical daily dose
- If the quantity was less than 7, the quantity was replaced to equal 30
- If the prescription duration was less than 7, the prescription duration was replaced to equal 30 days
- If quantity was less than the prescription duration then the prescription duration was changed to equal the quantity
- If the quantity was greater than 400, it was replace with the average quantity of other prescriptions for the same drug with the same strength
- The total duration was calculated as the sum of all prescription durations for each patient
- The total dosage was calculated as the sum of quantity multiplied by strength for all prescriptions in each patient
- The average daily dose was calculated as the total dosage divided by the total duration of therapy.
